# Hippocampal theta activity during encoding promotes subsequent associative memory in humans

**DOI:** 10.1093/cercor/bhad162

**Published:** 2023-05-09

**Authors:** Bárður H Joensen, Daniel Bush, Umesh Vivekananda, Aidan J Horner, James A Bisby, Beate Diehl, Anna Miserocchi, Andrew W McEvoy, Matthew C Walker, Neil Burgess

**Affiliations:** UCL Queen Square Institute of Neurology, UCL, London WC1N 3BG, United Kingdom; UCL Institute of Cognitive Neuroscience, UCL, London, WC1N 3AZ, United Kingdom; Department of Clinical Neuroscience, Karolinska Institute, Stockholm 17165, Sweden; Department of Psychology, Uppsala University, Uppsala 751 42, Sweden; Department of Neuroscience, Physiology and Pharmacology, UCL, London, WC1E 6BT, United Kingdom; UCL Queen Square Institute of Neurology, UCL, London WC1N 3BG, United Kingdom; Department of Psychology, University of York, York, YO10 5DD, United Kingdom; York Biomedical Research Institute, University of York, York, YO10 5DD, United Kingdom; UCL Queen Square Institute of Neurology, UCL, London WC1N 3BG, United Kingdom; UCL Institute of Cognitive Neuroscience, UCL, London, WC1N 3AZ, United Kingdom; Division of Psychiatry, UCL, London, W1T 7BN, United Kingdom; UCL Queen Square Institute of Neurology, UCL, London WC1N 3BG, United Kingdom; UCL Queen Square Institute of Neurology, UCL, London WC1N 3BG, United Kingdom; UCL Queen Square Institute of Neurology, UCL, London WC1N 3BG, United Kingdom; UCL Queen Square Institute of Neurology, UCL, London WC1N 3BG, United Kingdom; UCL Queen Square Institute of Neurology, UCL, London WC1N 3BG, United Kingdom; UCL Institute of Cognitive Neuroscience, UCL, London, WC1N 3AZ, United Kingdom; Wellcome Centre for Human Neuroimaging, UCL, London, WC1N 3AR, United Kingdom

**Keywords:** hippocampus, associative memory, intracranial EEG, MEG, theta oscillations

## Abstract

Hippocampal theta oscillations have been implicated in associative memory in humans. However, findings from electrophysiological studies using scalp electroencephalography or magnetoencephalography, and those using intracranial electroencephalography are mixed. Here we asked 10 pre-surgical epilepsy patients undergoing intracranial electroencephalography recording, along with 21 participants undergoing magnetoencephalography recordings, to perform an associative memory task, and examined whether hippocampal theta activity during encoding was predictive of subsequent associative memory performance. Across the intracranial electroencephalography and magnetoencephalography studies, we observed that theta power in the hippocampus increased during encoding, and that this increase differed as a function of subsequent memory, with greater theta activity for pairs that were successfully retrieved in their entirety compared with those that were not remembered. This helps to clarify the role of theta oscillations in associative memory formation in humans, and further, demonstrates that findings in epilepsy patients undergoing intracranial electroencephalography recordings can be extended to healthy participants undergoing magnetoencephalography recordings.

## Introduction

Oscillatory activity within the hippocampal-entorhinal system has long been hypothesized to play a critical role in cognitive function (Buzsáki and Moser 2013). In particular, the 6–10 Hz theta rhythm dominates the local field potential in the rodent hippocampus, a region known to be critical for spatial and episodic memory in humans (O’Keefe and Nadel 1979; Eichenbaum and Cohen 2004). Continuous hippocampal theta activity is observed whenever the animal is moving (Vanderwolf 1969) or engaged in memory-guided behavior (Aronov et al. 2017). Memory function has also been linked to the presence of theta rhythmicity, with disruption of hippocampal theta abolishing spatial learning (Winson 1978; McNaughton et al. 2006).

Electrophysiological studies have demonstrated that theta oscillations are also present during movement in the human hippocampus (Ekstrom et al. 2005; Jacobs et al. 2010; Bohbot et al. 2017), albeit at shorter duration and lower amplitude than the rodent equivalent (Jacobs 2014). Similarly, substantial evidence exists to demonstrate a relationship between theta activity in the hippocampus and memory formation in humans (Herweg et al. 2020). However, results regarding the precise relationship between hippocampal theta and successful memory formation are mixed, with evidence from studies using scalp electroencephalography (EEG) or magnetoencephalography (MEG) demonstrating that theta activity during encoding is positively correlated with later memory success (Backus et al. 2016; Hanslmayr et al. 2011; Klimesch et al. 1996; Osipova et al. 2006; Gruber et al. 2008; Guderian and Düzel 2005; Herweg et al. 2016; Staudigl and Hanslmayr 2013; but see also Guderian et al. 2009 and Addante et al. 2011 for pre-stimulus encoding theta and subsequent memory performance), whereas those using intracranial EEG (iEEG) recordings in large part demonstrating that decreases in hippocampal theta during encoding are predictive of later memory success (Sederberg et al. 2007; Lega et al. 2012; Long et al. 2014; Long and Kahana 2015; Lin et al. 2017; Fellner et al. 2019; Solomon et al. 2019).

The reasons for these discrepancies are perhaps manyfold (Herweg et al. 2020), but we reasoned that one important factor may be related to differences in memory paradigms and/or the type of memory being assessed. For instance, among studies using scalp EEG and MEG, theta activity during encoding has been associated with subsequent recognition (Osipova et al. 2006), recollection (Guderian and Düzel 2005; Gruber et al. 2008; Herweg et al. 2016), and item to context matching (Staudigl and Hanslmayr 2013). On the other hand, studies using iEEG have tended to focus on the recognition or recall of single, isolated words. This point is critical given the proposed role of the hippocampus in the explicit encoding and retrieval of associative memories (Squire and Zola-Morgan 1991; Mayes et al. 2007), whereas item-based memory, or recognition more generally, may also be supported by MTL regions outside the hippocampus, possibly reflecting a simple familiarity signal (Aggleton and Brown 1999; Diana et al. 2007).

Here we aimed to assess the role of hippocampal theta activity in subsequent associative memory. To do this, we used a task and memoranda specifically designed to promote associative binding (Horner and Burgess 2014), given the role of the hippocampus in this process (Marr 1971; McClelland et al. 1995; Cohen et al. 1999; Davachi 2006; Mayes et al. 2007). We collected iEEG and MEG recordings across 2 studies, focusing on later retrieval success for paired associates that were imagined interacting during encoding (to promote deeper and more elaborative associative binding), while aiming to equate task demands for patients (in the iEEG study) and healthy participants (in the MEG study). While iEEG allows for direct recordings from the hippocampus, affording greater spatial resolution, these recordings are obtained from clinical populations. By collecting MEG recordings from healthy participants performing the same task, we can also assess the translation of hippocampal theta effects to nonclinical populations.

## Materials and methods

### Intracranial electroencephalography (EEG)

#### Patients

Fourteen patients with drug-resistant epilepsy undergoing iEEG monitoring for clinical purposes were asked to perform an associative memory task similar to that used in Horner and Burgess (2014). Ethical approval was granted by the NHS Research Ethics Committee (15/LO/1783) and informed written consent was obtained from each patient. Four patients were excluded from the analyses for the following reasons: (i) insufficient trials to allow for comparisons of subsequent memory performance (defined as <2 trials in any of the subsequent memory performance conditions, *n* = 2) and (ii) only 1 or no electrode contacts located in the hippocampus (*n* = 2). Accordingly, 10 patients (4 male/6 female, 9 right-handed, with *M* age ± SD of 36.20 ± 8.59 years) were included in the analyses.

Preimplantation MRI and postimplantation CT scans were co-registered to identify electrode locations in the hippocampus (*M* ± SD number of contacts = 3.10 ± 1.45), amygdala (*M* ± SD number of contacts = 2.10 ± 0.99), and temporal neocortex (*M* ± SD number of contacts = 10.50 ± 2.99). Electrode implantation was unilateral in all patients and dictated by clinical requirements (see Table 1 for clinical and general details).

**Table 1:** Clinical and general details of the patient population.

		Number of contacts			Implanted hemisphere
Patient ID	Seizure onset zone	HPC	Amygdala	TNC	
1	L HPC	4	2	12	L
2	L anterior HPC	2	3	9	L
3	R medial temporal	3	0	15	R
4	R anterior frontal	2	2	8	R
5	L occipito-temporal	2	1	15	L
6	Could not be localized	2	3	13	L
7	R middle frontal	2	2	8	R
8	R frontal/insular	3	2	7	R
9	R frontal lobe	6	3	9	R
10	Left PC	5	3	9	L

*Note.* L, left; R, right; HPC, hippocampus; PC, posterior cingulate; TNC, temporal neocortex.

Depth EEG was recorded at 512 Hz (patient 1), 1,024 Hz (patients 2–7, 10), or 2,048 Hz (patients 8–9) using a Micromed SD long-term monitoring system (Micromed). The EEG signal was referenced against a common white matter contact that was located remotely from the suspected epileptogenic focus in each patient. Recordings made at a higher sampling rate were downsampled to 512 Hz, to match those recorded with the lowest sampling rate, before any analyses were performed.

#### Materials

The stimuli consisted of 36 locations (e.g. *kitchen*), 36 famous people (e.g. *Barack Obama*), 36 common objects (e.g. *hammer*), and 36 animals (e.g. *dog*). From these, 18 randomized location–person–object and 18 randomized location–person–animal events were generated for each patient. For each patient, 9 location-person-object and 9 location-person-animal events were then randomly assigned to be learnt during encoding, and elements from the remaining events were used as foils (i.e., ‘new’ cues) during the old/new recognition judgement at test (see below).

#### Task

**Fig. 1:**
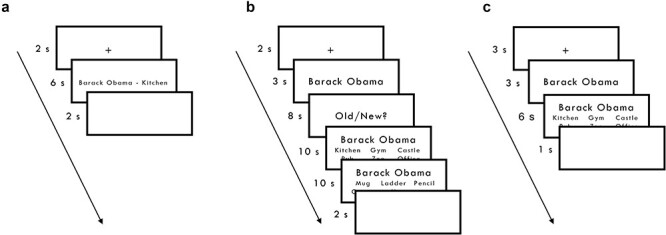
Experimental design. **a**) Encoding: participants saw multiple word pairs. Each presentation was preceded by a 2,000-ms fixation cross and followed by a 2,000-ms blank screen. **b**) Test (iEEG study): patients were presented with a single cue for 3 s and subsequently asked to indicate whether the cue was presented during encoding (i.e. old/new?). Patients had 8,000-ms to make a judgment. Patients were then required to retrieve one of the other elements from the same event as the cue from among 5 foils (elements of the same type from other events) in 10,000 ms. Both elements that were paired with the cue were tested in immediate succession of each other. Each test trial was preceded by a 2,000-ms fixation cross and followed by a 2,000-ms blank screen. **c**) Test (MEG study): participants were presented with a single cue for 3,000-ms and then required to retrieve one of the other elements from the same event from among 5 foils in 6,000 ms. Each test trial was preceded by a 3,000-ms fixation cross and followed by a 1,000-ms blank screen.

The memory task was similar to that developed by Horner and Burgess (2014). At encoding (see Fig. 1a), participants were separately presented with 3 overlapping pairs belonging to 18 events (e.g. *Barack Obama*–*kitchen*, *kitchen*–*hammer*, *hammer*–*Barack Obama* for the event *Barack Obama*, *kitchen*, *hammer*). All pairs were presented on a computer screen as text; 1 element to the left and 1 to the right of fixation. The left/right assignment was randomly chosen on each trial. Each word-pair remained on screen for 6,000 ms. Patients were instructed to imagine, as vividly as possible, the elements interacting in a meaningful way. The word-pair presentation was preceded by a 2,000-ms fixation and followed by a 2,000-ms blank screen.

The pairs were presented across 3 blocks with one pair from each event presented during each block, such that the presentation of a pair from one event was interleaved with the presentation of pairs from other events. Within each block, the presentation order of events was randomized. Furthermore, the order of presentations across the 3 blocks was pseudo-randomized such that the presentation order of 1/3 of the events was (i) person–location, location–object, object–person, (ii) location–object, object–person, person–location, and (iii) object–person, person–location, object–person, respectively.

During test (see Fig. 1b), patients were first presented with a cue (e.g. *Barack Obama*) that was drawn from one of the events learnt during encoding or from an equal number of novel events that patients had not seen during encoding. Each cue was presented in the center of the screen and remained on-screen for 3,000 ms. Patients were then asked to indicate whether this was an element that had been seen during encoding using an old/new recognition judgment. Patients had a maximum of 8,000 ms to provide a response. For “old” cues only, the old/new judgment was followed by a forced-choice associative memory task, irrespective of whether the recognition response was correct or incorrect. During the associative memory test, 6 possible associates (1 target and 5 foils) were presented alongside the cue element. The target would be one of the other elements seen together with the cue during encoding. The 5 foils would be elements from the same category as the target. For instance, if the patient had been presented with *Barack Obama*–*kitchen* during encoding and cued with *Barack Obama* during test, then the target would be *kitchen* and the 5 foils would be other randomly selected locations from other events seen during encoding.

For each cue, both elements that were paired with the cue during encoding would be tested in immediate succession before patients were presented with another cue element. For example, if a patient was presented with *Barack Obama* and required to retrieve *kitchen*, they would then be asked to retrieve *hammer* from among 5 randomly selected objects from other events. The target and foils were presented in 2 rows of 3 below the cue, and the location of the correct target element was randomly selected on each retrieval trial. Patients had a maximum of 10,000 ms to respond with a key press. Responses that fell outside this response window were treated as incorrect (*M* ± SD% of missing response = 4.91 ± 5.42).

Each event was tested with cue–target associations in both directions across 3 blocks. For instance, during block 1, patients could be cued with *Barack Obama* and asked to retrieve *kitchen* and then *hammer*, during block 2 cued with *kitchen* and required to retrieve *hammer* and then *Barack Obama*, and lastly during block 3 cued with *hammer* and asked to retrieve *Barack Obama*, then *kitchen*. Hence, for each event, each element acted as a cue once across the 3 blocks (e.g. block 1: *Barack Obama*; block 2: *kitchen*; block 3: *hammer*) and as a retrieval target twice across the 3 blocks (e.g. *Barack Obama*: blocks 2 and 3; *kitchen*: blocks 1 and 3; *hammer*: blocks 1 and 2).

Encoding and test were split into 2 phases, such that all 3 pairs from the first 9 events were encoded (making a total of 27 encoding trials) and then tested in both directions (making a total of 54 retrieval trials), before pairs from the remaining 9 events were encoded and tested. Hence, across the 2 encoding/test phases, patients were presented with a total of 54 encoding trials, 108 old/new recognition trials, and 108 associative memory retrieval trials. Note that retrieval trials followed only after the presentation of cue elements that patients had seen during encoding. As such, during test, patients were required to make a total of 108 old/new recognition judgments; 54 of which related to elements presented during encoding (e.g. *Barack Obama*) and were followed by 2 associative retrieval trials (e.g. retrieve *kitchen*, then *hammer*); and 54 of which related to elements that patients had not seen during encoding and were not followed by any associative retrieval trials.

Each test trial (composed of a cue presentation, old/new judgment, and 2 associative retrieval trials, when applicable) was preceded by a 2,000-ms fixation and followed by a 2,000-ms blank screen.

#### iEEG time-frequency analysis

Estimates of oscillatory power during the encoding period were obtained by convolving the EEG signal with a 7-cycle Morlet wavelet generated using SPM12 (Litvak et al. 2011). Time-frequency data were extracted from 2,000 ms before the start of each encoding trial to 2,000 ms after the end of the encoding period. Power values were obtained, separately for each encoding trial, for 55 logarithmically spaced frequencies in the 2–82 Hz range, and log transformed before mean power in each band between 1,000 and 500 ms prior to the start of the encoding period was subtracted from the data at all other time points to give a measure of power change from baseline in each frequency band. Data from the time windows before the baseline period (−2,000 to −1,000 ms) and after (6,000–8,000 ms) the encoding period were then discarded. Finally, all trials that had visually identified interictal spikes within the time window used for convolution were excluded prior to averaging over electrode contacts (*M* ± SD% of excluded trials = 12.87 ± 16.53; *M* ± SD number of included trials per condition = 15.80 ± 11.34, 16.99 ± 4.08, 14.25 ± 9.37, for 0, 1, and both directions correct, respectively).

### Magnetoencephalography

#### Participants

Twenty-six participants were recruited to perform the associative memory task. Ethical approval was granted by the local research ethics committee at University College London and all participants gave written informed consent to take part. All participants were compensated for their participation. Five participants were excluded from the analyses for the following reasons: (i) poor data quality (*n* = 1) and (ii) insufficient trials to allow for comparisons of subsequent memory performance (again, defined as <2 trials in any of the subsequent memory performance conditions, *n* = 4). Thus, a total of 21 participants (4 male/17 female, 21 right-handed, with a *M* age ± SD of 24.10 ± 2.97 years) were included in the analyses.

#### Materials

The stimuli consisted of 36 locations (e.g. *kitchen*), 36 famous people (e.g. *Barack Obama*), 36 common objects (e.g. *hammer*), and 36 animals (e.g. *dog*). For each participant, 18 of the 4-element sets were randomly assigned to a *closed-loop* associative structure. For closed-loops, 9 of the 4-element sets were assigned to be location–person–object events, and the remaining 9 were location–person–animal events. The remaining 18 4-element sets were randomly assigned to an *open-loop* associative structure, all of which contained all 4 elements.

#### Task

The memory task was identical to that used in the iEEG data set, with the following exceptions. During encoding, participants learnt 3 overlapping pairs from 36 events that formed either a closed- or open-loop associative structure (Horner and Burgess 2014). Closed- and open-loops differ in that all 3 elements of an event (e.g. *Barack Obama*, *kitchen*, *hammer*) are presented paired with all other elements of the same event (e.g. *Barack Obama*–*kitchen*, *kitchen*–*hammer*, *hammer*–*Barack Obama*) for closed-loops, whereas 4 elements belonging to the same event (e.g. *David Beckham*, *office*, *wallet*, *lion*) are presented as a chain of overlapping pairs (e.g. *David Beckham*–*office*, *office*–*wallet*, *wallet*–*lion*) for open-loops. This experimental manipulation has previously been shown to produce greater evidence of pattern completion (i.e. the retrieval of all elements of an event when presented with a single element as a cue) for closed- compared with open-loops, both in terms of behavioral responses and BOLD activity (Horner and Burgess 2014; Horner et al. 2015; Grande et al. 2019; Joensen et al. 2020). In the iEEG study, only 18 closed-loop events were used to avoid overtaxing the patients, and to focus on those events most likely to provide evidence of associative memory. Here, we were primarily interested in whether theta oscillations during the encoding of a given pair are predictive of later retrieval successes for that same pair, so we do not distinguish between these 2 types of associative structure in the MEG analyses. However, we have included a Supplementary section comparing subsequent memory effects between closed- and open-loop events in the MEG study (no differences were found).

At test (see Fig. 1c), participants were not required to make an old/new judgment as in the iEEG study. Instead, cue presentation was immediately followed by a forced-choice retrieval trial where participants were required to select the element that was previously paired with the cue from 6 possible target elements. Participants had a maximum of 6,000 ms to respond with a key press during each test trial. Responses that fell outside this response window were treated as incorrect (*M* ± SD% of missing response = 1.83 ± 2.37). Each of the 36 events were tested with cue–target associations in both directions across 6 retrieval blocks, with one randomly chosen pair from each event tested in each block, making a total of 216 retrieval trials. Each test trial (composed of a cue presentation and retrieval trial) was preceded by a 3,000-ms fixation and followed by a 1,000-ms blank screen.

Note also that in contrast to the iEEG study, encoding and test were not split into 2 encoding/test phases. Instead, participants encoded pairs belonging to all 36 events prior to being tested on all cue–target associations. Again, this difference stems from the fact that the iEEG task was designed to avoid overtaxing the patients.

#### MEG source power analysis

MEG recordings were made using a 275-channel Canadian Thin Films MEG system with SQUID-based axial gradiometers (VSM Med-TECH) while participants sat upright in a magnetically shielded room. Recordings were made at a sampling rate of 480 Hz. Head position coils were attached to nasion and left and right preauricular sites for anatomical coregistration.

All data preprocessing and analyses were performed in SPM12 (Litvak et al. 2011) with the only exception being that eye blink and heartbeat artifacts were identified and removed using independent component analysis, implemented in Fieldtrip (Oostenveld et al. 2011) and EEGLAB (Delorme and Makeig 2004). High-pass (0.1 Hz) and notch (48–52 Hz) filters were applied to the data to remove drift and line noise, respectively, and the data were epoched from 2,000 ms prior to the onset of the encoding period to 2,000 ms following the end of the encoding period.

MEG source localization was conducted using the linearly constrained minimum variance beamformer with a single-shell forward model to generate maps of mean source power differences (Barnes and Hillebrand 2003) for trials where participants were presented with pairs that they subsequently failed to retrieve, successfully retrieved in 1 direction but not the other, and those where they successfully retrieved the pairs in both directions (i.e. 0, 1, and both directions correct). Trials containing muscle artifacts were visually identified and removed prior to source localization (*M* ± SD% of excluded trials = 5.78 ± 8.10; *M* ± SD number of included trials per condition = 20.00 ± 16.73, 25.39 ± 5.75, 56.48 ± 23.09, for 0, 1, and both directions correct, respectively). Maps were generated on a 10-mm grid, coregistered to MNI coordinates and all SPM images were smoothed using a 12 × 12 × 12-mm full-width half-maximum Gaussian kernel.

Given our specific hypothesis regarding the hippocampus, hippocampal SPM results are small volume corrected (SVC) within a bilateral hippocampal mask (Fig. 4a). The mask was generated using WFU PickAtlas (Maldjian et al. 2003) with hippocampal regions defined from the Automated Anatomical Labelling atlas (Tzourio-Mazoyer et al. 2002). For completeness, we also present results from outside the hippocampus. All effects reported from outside the hippocampus are *P*_FWE_ < 0.05 whole-brain corrected.

## Results

### Behavior

Our associative memory task involved patients/participants first encoding a series of pairwise associations (e.g. *Barack Obama*–*kitchen*). Patients/participants were then required to retrieve the learnt pairs using a forced-choice associative memory test, where a cue element (e.g. *Barack Obama*) was presented alongside 6 possible targets (e.g. *kitchen* and 5 foils from the same category as the target). Each cue–target association was retrieved in both directions (e.g. cue: *Barack Obama*, target: *kitchen*; cue: *kitchen*, target: *Barack Obama*).

The mean proportion correct and mean proportion of pairs retrieved correctly in 0, 1, and both directions in the iEEG and MEG study are presented in Table 2. In the MEG study, mean memory performance was 68%, which is comparable to performance in previous studies using a similar paradigm (Horner and Burgess 2014; Horner et al. 2015) and significantly above chance (~16.7%), *t*(20) = 12.12, *P* < 0.001, *d* = 2.65. Mean memory performance in the iEEG study was 48%, which is numerically lower than that seen in prior studies with healthy participants. This is consistent with evidence showing decreases in memory performance in patients with focal epilepsy (Delaney et al. 1980), but we note that patients were still able to retrieve the learnt pairs at a level well above chance, *t*(9) = 5.26, *P* < 0.001, *d* = 1.66. Similarly, patients’ old/new recognition performance (hits: *M* = 0.92, SD = 0.04; false alarms: *M* = 0.04, SD = 0.05; *d*’: *M* = 3.30, SD = 0.57) was significantly above chance (0.50), *t*(9) = 18.25, *P* < 0.001, *d* = 5.78. Note that for patients who had no false alarms (*n* = 4), values were set to 1/*N*, where *N* is the number of trials corresponding to “new” cues.

**Table 2:** Mean proportion correct (and standard deviations) and mean proportion of pairs retrieved correctly in 0, 1, and both directions (and standard deviations) in the iEEG and MEG data.

	Proportion correct	Zero direction correct	One direction correct	Both directions correct
iEEG	0.48 (0.19)	0.33 (0.21)	0.37 (0.05)	0.30 (0.18)
MEG	0.68 (0.19)	0.20 (0.17)	0.25 (0.06)	0.55 (0.21)

### iEEG study

#### Theta activity in hippocampal contacts and subsequent memory

**Fig. 2:**
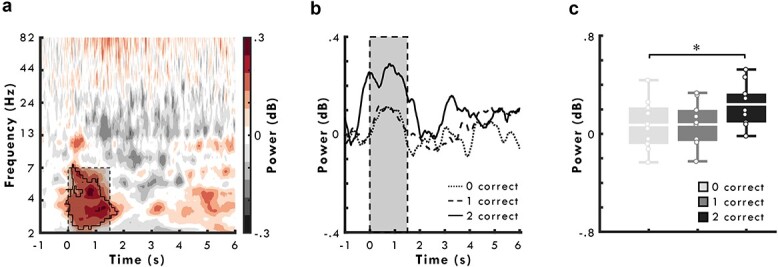
Theta power on hippocampal iEEG contacts. **a**) Time-frequency power spectrogram, over hippocampal contacts, from −1,000 ms prior to 6,000 ms after the onset of the encoding period. **b**) Time series plot of mean theta power (2–7 Hz) over hippocampal contacts split by subsequent memory performance from −1,000 ms prior to 6,000 ms after the onset of the encoding period. **c**) Boxplot of mean theta power (2–7 Hz) between 0 and 1,500 ms after the onset of encoding (shaded dashed box in **a**) and **b**)), over hippocampal contacts, split by later memory performance. Lines in boxplot represent mean power. Bottom and top edges of boxes indicate the 25th and 75th percentiles, respectively. Whiskers represent minimum and maximum data points. Overlaid dots represent individual data points. ^*^P < 0.05.

As a first step, we looked for increases in oscillatory power on hippocampal electrode contacts during the encoding period. For this analysis, we used a Monte Carlo cluster analysis approach (Maris and Oostenveld 2007) implemented in Fieldtrip (Oostenveld et al. 2011) to identify time- and frequency-bands (*α* = 0.05 (1-tailed), #permutations = 1,000) where power increased relative to baseline. Note that because the number of contacts differed across patients, power values were averaged over electrode contacts for each patient prior to analysis. This revealed a significant positive cluster in the theta frequency band (at ~2–7 Hz) from ~0 to 1,500 ms following the onset of the encoding period (*t_sum_* = 2.46 × 10^4^, *t_max_* = 4.87, *t_mean_* = 2.59, *P* = 0.03, *d* = 0.82; see outlined cluster in Fig. 2a), demonstrating that theta oscillations in the hippocampus are engaged during encoding.

Next, to establish whether increases in hippocampal theta power were predictive of subsequent memory, we asked whether theta power (at 2–7 Hz between 0 and 1,500 ms; see shaded dashed box in Fig. 2a) differed between the encoding of pairs that were consistently retrieved correctly in both directions and those that patients consistently failed to retrieve. A paired sample *t*-test revealed that theta power was significantly greater during the encoding of pairs that were remembered correctly in both vs 0 directions, *t*(9) = 2.68, *P* = 0.03, *d* = 0.85 (Fig. 2c, see also Fig. 2b for the time profile of mean theta power over the entire encoding period; note that this 2-tailed significance value is not corrected for multiple comparisons, being our main effect of interest).

For completeness, we also performed a 1-way ANOVA comparing mean theta power (at 2–7 Hz between 0 and 1,500 ms) during the encoding of pairs that were subsequently retrieved correctly in 0 vs 1 vs both directions. It is important to note, however, that activity relating to pairs that were later retrieved correctly in only 1 direction is not a good indicator of subsequent memory, as it reflects a mixture of both successful and unsuccessful encoding. This ANOVA did not reveal a significant effect of subsequent memory, *F*(2, 27) = 2.70, *P* = 0.09, *η_p_*^2^ = 0.17. Similarly, paired sample *t*-tests comparing theta power between pairs that patients did not later retrieve vs those remembered in 1 direction and those remembered in 1 vs both directions revealed no significant effects, *t*s < 1.93, *P*s > 0.08, consistent with the indeterminate status of pairs correctly retrieved in only 1 direction. In sum, we see evidence to suggest that theta oscillations during encoding contribute to later memory, at least in so far as theta activity differs between pairs that were recalled in their entirety and those that patients did not remember.

Interestingly, some prior studies have shown that pre-encoding theta activity can also be predictive of subsequent memory success (Otten et al. 2006; Guderian et al. 2009; Fell et al. 2011). However, we found no significant relationship between raw theta activity (in the 2–7 Hz band) averaged over the −1,000 to −500-ms pre-encoding baseline window (used in the analyses above) and subsequent memory (i.e. between pairs that patients retrieved in 0, 1, or both directions correctly), *t*s < 1.70, *P*s > 0.12.

#### Spectral tilt on hippocampal contacts

Having examined the time-frequency spectrograms between encoding and baseline, and across differences in subsequent memory, we now wanted to ascertain whether these differences may be affected by changes in spectral “tilt” (Fellner et al. 2019). To do so, we used the irregular-resampling auto-spectral analysis method (Wen and Liu 2016), implemented in Fieldtrip (Oostenveld et al. 2011), to separate the background “fractal” and oscillatory components of the EEG signal during the 0–1,500-ms encoding period of interest. We then compared power in the theta frequency band identified above between the fractal power spectrum across trials corresponding to the encoding pairs that participants retrieved correctly in 0, 1, or both directions, but found no main effect of subsequent memory success, *F*(2, 18) = 0.09, *P* = 0.91, *η_p_*^2^ < 0.01.

For completeness, we also compared power in the fractal power spectrum between trials corresponding to the encoding of pairs that patients retrieved correctly in 0 directions and those retrieved correctly in both directions using a paired sample *t*-test, but observed no effect of subsequent memory success, *t*(9) = 0.44, *P* = 0.67, *d* = 0.13. In sum, this suggests that there is no change in spectral tilt during the time window of interest according to subsequent memory performance and is consistent with the proposal that while theta activity supports associative memory, spectral tilt may reflect a more general index of activation (Fellner et al. 2019; Herweg et al. 2020).

#### Theta activity across other temporal lobe contacts

As theta oscillations have been shown to be widespread across the temporal lobe during encoding (Sederberg et al. 2003), we next assessed whether memory-related changes in theta activity were restricted to hippocampal electrode contacts or if they extended to other temporal lobe contacts. As a first step, we performed 2 separate cluster analyses (*α* = 0.05 (1-tailed), #permutations = 1,000), to identify increases in oscillatory power on electrode contacts in the temporal neocortex or amygdala during encoding. Note that 1 patient had no depth electrodes located in the amygdala and as such this analysis includes only 9 patients (see Table 1). No significant clusters were observed in the temporal neocortex (*P*s > 0.15). However, a significant cluster was observed in the amygdala, where power increased relative to baseline (at ~3–6 Hz) at ~500–2,000 ms following the onset of encoding (*t_sum_* = 1.39 × 10^4^, *t_max_* = 4.42, *t_mean_* = 2.67, *P* = 0.04, *d* = 0.94).

To examine whether power during encoding in the amygdala differed according to subsequent memory performance, we assessed whether increases in theta power (at 3–6 Hz between 500 and 2,000 ms) differed between the encoding of pairs that were retrieved correctly in both directions and those that were consistently not retrieved. A paired sample *t-*test revealed no significant difference, *t*(8) = 0.81, *P* = 0.94, *d* = 0.03. Similarly, paired sample *t*-tests comparing theta power between pairs that were not later retrieved and those remembered in only 1 direction and those remembered in 1 and both directions revealed no significant effects, *ts* < 0.19, *Ps* > 0.85.

**Fig. 3:**
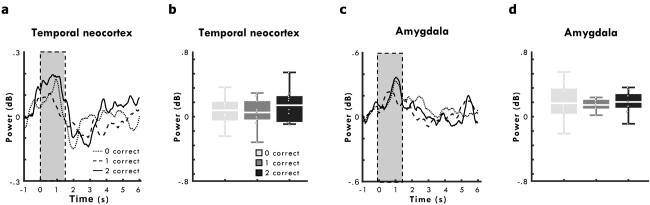
Theta power in temporal neocortex and amygdala iEEG contacts. **a**, **b**) Temporal neocortex contacts. **a**) Time series plot of mean theta power (2–7 Hz), split by subsequent memory performance, from −1,000 ms prior to 6,000 ms after the onset of the encoding period. **b**) Boxplot of mean theta power (2–7 Hz) between 0 and 1,500 ms after the onset of encoding (dashed box in **a**)), split by later memory performance. **c**, **d**) Amygdala contacts. **c**) Time series plot of mean theta power (2–7 Hz), split by subsequent memory performance, from −1,000 ms prior to 6,000 ms after the onset of the encoding period. **d**) Boxplot of mean theta power (2–7 Hz) between 0 and 1,500 ms after the onset of encoding (dashed box in **c**)), split by later memory performance. Lines in boxplots represent mean power. Bottom and top edges of boxes indicate the 25th and 75th percentiles, respectively. Whiskers represent minimum and maximum data points. Overlaid dots represent individual data points.

Next, we contrasted mean power in the time (0–1,500 ms) and frequency (2–7 Hz) band where hippocampal theta activity increased relative to baseline during encoding, split by subsequent memory performance (i.e. 0 vs 1 vs both directions correct), for electrode contacts located in the amygdala and temporal neocortex. Pairwise comparisons of theta power split by subsequent memory revealed no significant differences in mean theta activity (at 2–7 Hz between 0 and 1,500 ms) in contacts located in the temporal neocortex, *t*s < 1.41, *P*s > 0.19 (Fig. 3b), or amygdala, *t*s < 1.06, *P*s > 0.31 (Fig. 3d).

These results suggest that changes in theta power as a function of subsequent associative memory may show a different pattern within the hippocampus compared with that in the amygdala and temporal neocortex. However, for the 9 patients with electrode contacts in all 3 regions, a 2x3 ANOVA for subsequent memory (0 vs 2 directions correct) and region (hippocampus vs temporal neocortex vs amygdala) showed that theta power differed significantly according to subsequent memory performance, *F*(1, 8) = 6.43, *P* = 0.04, *η_p_*^2^ = 0.45, but not across electrode contacts located in the hippocampus, temporal neocortex and amygdala, *F*(2, 16) = 1.23, *P* = 0.32, *η_p_*^2^ = 0.13, even as an interaction with subsequent memory performance, *F*(2, 16) = 0.83, *P* = 0.45, *η_p_*^2^ = 0.09.

To assess this further, we examined the relationship between trial-by-trial variations in mean theta power in the 0 to 1500-ms encoding window within each region. To do so, we first computed power for each electrode contact in each region and then estimated the linear relationship between mean theta power (at 2–7 Hz) on each contact across the regions. We then averaged the beta coefficients for each pair of electrode contacts across the regions and assessed whether the observed fit consistently deviated from 0 using 1-sample *t*-tests.

This analysis revealed that trial-by-trial theta power in the hippocampus was significantly correlated with theta power in both temporal neocortex, *t*(9) = 4.99, *P* < 0.001, *d* = 1.58, and amygdala, *t*(9) = 4.86, *P* < 0.01, *d* = 1.62. Combined, these findings are consistent with the idea that theta oscillations across the temporal lobe may be driven by a single source (Bush et al. 2017), with subsequent memory effects being most pronounced in the hippocampus but also present—to some extent—in other regions (given the main effect of subsequent memory in the 2 × 3 ANOVA above).

### MEG study

#### Hippocampal theta power and subsequent memory

To corroborate our intracranial hippocampal effects, we examined whether theta power in the source reconstructed MEG data during encoding was predictive of differences in subsequent memory performance. We focused this analysis on the theta frequency band (2–7 Hz) and time window (0–1,500 ms following the onset of encoding) identified in the iEEG study. However, as a control to ascertain whether any effect was specific to the theta frequency band, we also assessed power changes in 3 other canonical frequency bands (i.e. alpha: 8–12 Hz, beta: 13–29 Hz, and gamma: 30–80 Hz). Note that the method for computing mean source power used here (Litvak et al. 2011) requires that the baseline and time window of interests are of equivalent duration. Therefore, all source power values reflect differences in mean theta power at 0–1,500 ms following the onset of encoding relative to 2,000–500 ms prior to the onset of the encoding period (as compared with the 1,000–500-ms pre-encoding baseline used in the iEEG study).

As a first step, source reconstructed theta power was estimated separately for trials associated with pairs that participants later failed to retrieve, retrieved correctly in 1 direction, and those retrieved correctly in both directions. To assess our main effect of interest, source reconstructed theta power was examined in a second-level general linear model that contained a single *t*-contrast between pairs retrieved correctly in 0 and both directions. This analysis revealed an effect of subsequent memory with greater theta activity within the bilateral hippocampal mask for trials later retrieved correctly in both directions than those not retrieved correctly, with a maxima around the right hippocampus (28, −4, −28; *Z* = 3.17, *P*_FWE/SVC_ = 0.03). In addition, there was an effect of subsequent memory at the whole-brain cluster corrected level in the inferior temporal (−52, −56, −24; *Z* = 6.52, *P*_FWE_ < 0.01) and cingulate gyrus (8, 24, 16; *Z* = 4.65, *P*_FWE/SVC_ < 0.01). No such differences were seen when contrasting source reconstructed theta power for trials associated with pairs retrieved in 0 and 1 directions correctly and those retrieved correctly in 1 and both directions. Similarly, no differences were seen in the alpha, beta, or gamma frequency bands, either at the whole-brain level or within the bilateral hippocampal mask.

**Fig. 4:**
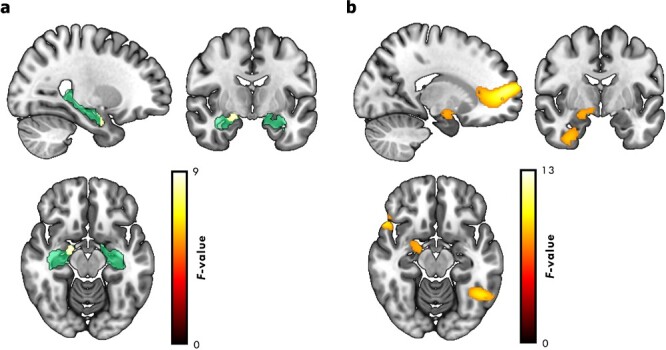
Source-localized MEG theta power. **a**) Source-localized theta power effect of subsequent memory performance (visualized at *P*_FWE/SVC_ < 0.05) within the bilateral hippocampal mask (green outline) used for small-volume correction. **b**) Source localized theta power effect of subsequent memory performance (visualized at an uncorrected threshold of *P* < 0.001) across the whole brain.

For consistency with the iEEG study, we next compared estimates of source reconstructed theta power between pairs that were later retrieved correctly in 0, 1, and both directions in a second level general linear model. This model contained a single *F*-contrast corresponding to the main effect of subsequent memory. This analysis also revealed an effect of subsequent memory within the bilateral hippocampal mask, with a maxima around the right hippocampus (18, −6, −14; *Z* = 3.22, *P*_FWE/SVC_ = 0.03; Fig. 4a). In addition, there was a single subsequent memory effect at the whole-brain corrected level in the medial prefrontal cortex (10, 22, 18; *Z* = 4.05, *P*_FWE_ = 0.04; Fig. 4b). No such effects were seen in the alpha, beta, or gamma frequency bands, either on a whole-brain level or within the bilateral hippocampal mask.

To assess this hippocampal subsequent memory effect further, we extracted mean source power for all encoding trials split by subsequent memory from a 10-mm sphere centered on the peak right hippocampal voxel showing a consistent main effect of subsequent memory (Fig. 4a).

**Fig. 5:**
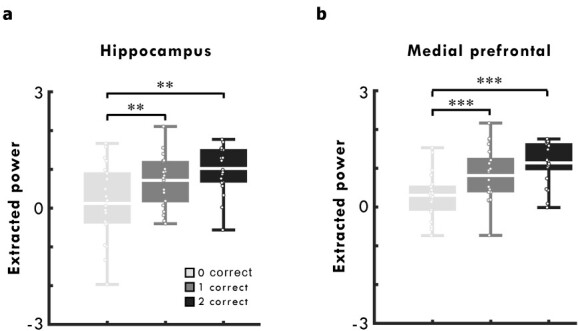
Subsequent memory effects. **a**) Boxplot of mean hippocampal power (extracted from a 10-mm sphere centered on the peak hippocampal voxel), split by later memory performance. **b**) Boxplot of mean medial prefrontal power (extracted from a 10-mm sphere centered on the peak prefrontal voxel), split by later memory performance. Lines in boxplot represent mean power. Bottom and top edges of boxes indicate the 25th and 75th percentiles, respectively. Whiskers represent minimum and maximum data points. Overlaid dots represent individual data points. ^*^^*^P < 0.01, ^*^^*^^*^P < 0.001.

A paired sample *t*-test showed that extracted theta power was greater for encoding trials associated with pairs remembered in both directions relative to those that participants failed to retrieve, *t*(20) = 3.57, *P* < 0.001, *d* = 0.78 (Fig. 5a; note that the significance value is not corrected for multiple comparisons as this was our main effect of interest). Interestingly, in contrast to the iEEG study, a paired sample *t*-test also showed that theta power for pairs remembered in 1 direction was greater than for pairs that participants did not later remember, *t*(20) = 3.38, *P* < 0.01, *d* = 0.74 (Fig. 5a). There was no difference between encoding trials associated with pairs remembered in 1 relative to both directions, *t*(20) = 1.74, *P* = 0.10, *d* = 0.38.

Similar effects were also seen when mean source power was extracted from a 10-mm sphere centered on the peak medial prefrontal voxel (Fig. 4b), with greater theta power for encoding trials associated with pairs remembered in both direction, *t*(20) = 6.01, *P* < 0.001, *d* = 1.31, and 1 direction, *t*(20) = 4.24, *P* < 0.001, *d* = 0.92, relative to those pairs that were not remembered, respectively (Fig. 5b).

We next assessed whether raw theta activity in the source reconstructed MEG data during the −2,000 to −500-ms baseline period differed between encoding trials that were associated with pairs retrieved correctly in 0, 1, or both directions. To do this, we computed mean source power in the 2–7 Hz theta frequency range during that baseline period, and then extracted power values from the right hippocampal-centered 10-mm sphere specified above. Pairwise comparisons of the extracted power revealed that theta activity was significantly lower for trials associated with pairs that were retrieved correctly in 1 direction relative to those that participants failed to retrieve, *t*(20) = 2.33, *P* = 0.03, *d* = 0.51. This difference may contribute to the theta power effect seen when contrasting pairs retrieved correctly in 1 and 0 directions in the baseline normalized MEG data during the encoding period. No other significant differences during the baseline period were seen, *t*s < 1.82, *P*s > 0.09.

## Discussion

In humans, hippocampal theta is thought to be critical for successful memory formation (Buzsáki 2002). However, findings regarding the precise contribution of theta oscillations to successful encoding and subsequent memory are mixed. Here, we used an associative memory paradigm that required patients and participants to vividly imagine pairs of elements interacting (Horner and Burgess 2014), combined with iEEG and MEG recordings, to assess the contribution of hippocampal theta activity during encoding to later associative memory performance. In the iEEG study, we showed that theta activity increased during encoding, and that this increase was greater for pairs that were subsequently retrieved successfully in both directions relative to those that were not remembered at all. In the MEG study, we corroborated these findings, demonstrating that the difference between theta activity for pairs remembered in both directions and those that participants failed to retrieve translated to healthy populations.

Investigations of the role of theta activity in memory formation have yielded contrasting results, with studies using non-invasive recordings in healthy populations showing that increased theta during (e.g. Osipova et al. 2006; Gruber et al. 2008) or prior to (e.g. Guderian et al. 2009; Addante et al. 2011) encoding is associated with later memory success, whereas intracranial studies have, in large part, demonstrated that decreases in theta activity during encoding contribute to subsequent memory performance (e.g. Long et al. 2014; Solomon et al. 2019). We speculated that these differences may, at least partially, arise from differences in memory paradigms or the type of memories being examined, as studies using iEEG recordings have tended to focus on the recognition or free recall of single, isolated items. We aimed to address that discrepancy here by also assessing the role of theta encoding activity in item recognition in the iEEG study. However, patients’ recognition performance was too high to allow for meaningful comparisons between correct and incorrect recognition (i.e. hits and misses). As such, further work is needed to address this possibility, but we note that intracranial studies that have correlated encoding activity to later associative memory, rather than item memory, have shown that increased theta power at encoding is positively related to later memory performance (Miller et al. 2018; Kota et al. 2020).

The associative nature of the task used here, along with requirements to vividly imagine the items interacting, might be important factors in our finding of positive subsequent memory theta effects in the iEEG and MEG studies, as compared with the less deliberative free recall of single items used elsewhere when negative theta subsequent memory effects have been observed (e.g. Long et al. 2014). It is possible that the encoding demands in this study encourage more contextually rich and/or high confidence retrieval states, which contributes to our subsequent memory contrast (e.g. Staudigl and Hanslmayr 2013). However, we cannot rule out the possibility that the positive hippocampal theta subsequent memory effects observed here, and negative hippocampal theta subsequent memory effects observed elsewhere reflect separate effects, each of which contribute to later memory (Long et al. 2014).

We have also demonstrated that our intracranial results extend to a measure of oscillatory activity recorded using MEG in nonclinical populations. This point is critical because human iEEG studies face the issue of extrapolating findings in patients to the general population, and here we show that averaged effects across patient populations can, at least in this instance, be translated to healthy participants. Although we were able to detect the presence of positive hippocampal theta subsequent memory effects in both the iEEG and MEG study, we are unable to definitively confirm that these effects originate in the hippocampus. Nonetheless, the observation of correlated trial-by-trial variations in theta activity across temporal lobe recording sites in the iEEG study support the proposal that this activity may be driven by a single source (Bush et al. 2017). In addition, our MEG source localization results, and the fact that subsequent memory effects in the iEEG study only reached significance on hippocampal electrode contacts, suggest the hippocampus as the most likely origin.

Although the findings in the MEG study in large part overlapped with those observed in the iEEG study, they did differ in one aspect. In the iEEG study, we observed that hippocampal theta power during encoding was greater for pairs subsequently retrieved correctly in both directions relative to those not retrieved at all (a finding replicated in the MEG study). However, in the MEG study (when extracting mean theta power from the hippocampal region with the peak subsequent memory effect), we also saw that theta power was greater for those pairs that were retrieved correctly in 1 direction but not the other, compared with those that participants did not subsequently remember. It is possible that epileptic pathology and the relatively small sample size in the iEEG study may have reduced our ability to detect such a difference. Interestingly though, when extracting mean theta power from the same region, we did observe that baseline theta activity in the MEG study was lower for pairs that participants later retrieved correctly in 1 direction relative to those that participants did not remember (an effect not seen in the iEEG study). It is possible that this difference during the baseline period may contribute to the theta power difference seen in the baseline corrected MEG data during encoding.

Indeed, we cannot rule out some contribution of baseline decreases in theta power to the positive subsequent memory effect we observed here. Except for the above, we saw that baseline activity was not predictive of subsequent memory performance. Nonetheless, even small, nonsignificant, variations in baseline theta activity may lead us to overestimate differences in encoding activity across pairs retrieved correctly in 0, 1, or both directions. In this sense, the results presented here could potentially reflect changes in some *system state* prior to encoding, in addition to changes induced by the presentation of the pairs or those evoked by the underlying theta rhythm.

We are also unable to specify the functions contributing to subsequent memory, which may include effects of attention or task engagement. Here we assess the contribution of hippocampal theta power to later memory performance by contrasting encoding trials associated with pairs that were correctly retrieved to those in which they were not. As such, our main effect of interest inevitably reflects activity related to the successful formation of associative memories, including activity that is not specific to mnemonic encoding, such as attention or task engagement. Future studies should aim to isolate the neural processes related to successful associative memory formation by controlling for the influence of attention and/or task engagement.

Finally, we note that, in contrast to our findings, some previous studies have also described theta subsequent memory effects in medial and lateral temporal lobe regions outside of the hippocampus (e.g. Hanslmayr et al. 2011). Although we have shown that theta power across temporal lobe recording sites was significantly correlated on a trial-by-trial basis, we did not find strong evidence for subsequent memory effects in these other regions. However, it is possible that our sensitivity to such an effect was reduced by greater variability in electrode placement in the temporal neocortex, relative to the hippocampus, and by averaging our results across contacts within each region for each patient prior to analyses. It is also possible that differences in task demands could account for these discrepancies. For instance, Greenberg et al. (2015) observed that theta power decreases in the temporal lobe (including the temporal neocortex) during encoding were predictive of subsequent memory performance for lists of word-pairs. In contrast, we required participants to richly imagine the paired associates interacting to promote cross-modal, associative binding, which is known to depend on the hippocampus (Marr 1971; McClelland et al. 1995; Cohen et al. 1999; Davachi 2006; Mayes et al. 2007).

In summary, across 2 complementary studies using iEEG and MEG recordings, we have shown that theta activity during encoding in the human hippocampus promotes subsequent associative memory. Importantly, we are able to demonstrate that hippocampal theta effects observed in patients in the iEEG study extrapolate to healthy participants.

## Supplementary Material

Joensen-et-al_CerebCortex_SupplMaterial_bhad162

## Data Availability

Analysis code and materials are available on the Open Science Framework (https://osf.io/5afsb/). Anonymized data can be shared by reasonable request from any qualified investigator.
