# Supplementary material for: Hippocampal theta activity during encoding promotes subsequent associative memory in humans
Source: Cereb Cortex. 2023 May 9;33(13):8792–802. doi: 10.1093/cercor/bhad162 (PMC10321091; doi:10.1093/cercor/bhad162)
Supplement: Joensen-et-al_CerebCortex_SupplMaterial_bhad162 [file joensen-et-al_cerebcortex_supplmaterial_bhad162.docx]

## Oscillatory power does not predict later memory for other pairs from the same event

In the MEG data, participants learnt 36 events, 18 of which formed closed-loops (as in the iEEG data) and open-loops, respectively. The critical difference between these two associative structures is that for closed-loops all three elements of an event (e.g., *Barack Obama*, *kitchen*, *hammer*) are presented paired with all other elements of the same event (e.g., *Barack* *Obama*-*kitchen*, *kitchen*-*hammer*, *hammer*-*Barack Obama*) at encoding, while for open-loops four elements belonging to the same event (e.g., *David Beckham*, *office*, *wallet*, *lion*) are presented as a chain of overlapping pairs (e.g., *David Beckham*-*office*, *office*-*wallet*, *wallet*-*lion*).

In previous work (Horner et al., 2015) we showed that BOLD activity in the hippocampus during the encoding of the third (i.e., final) pairwise association of a closed-loop (e.g., *hammer*-*Barack Obama*) is predictive of later memory for pairs learnt during the first and second encoding trials (i.e., *Barack Obama*-*kitchen*, *kitchen*-*hammer*). That is, BOLD activity during this encoding trial was predictive of later memory for the first and second encoded pairs from the same event when contrasting closed- vs open-loops. Critically, this effect was specific to the third encoding trials, and not the first and second encoding trials.

For consistency with prior work (Horner et al., 2015), we assessed whether oscillatory activity during the encoding of the first, second, and third pairs was predictive of subsequent memory performance for the other pairs from the same event. To do this, we regressed oscillatory power for each encoding trial with later memory performance for the other pairs from the same events. For example, if the trial corresponded to the encoding of *Barack Obama*-*kitchen*, then the value of the regressor would be the sum of later performance on retrieval trials assessing *kitchen*-*hammer* and *hammer*-*Barack Obama*. Note that because each pair was tested in both directions, the sum of memory performance ranges from zero to four. Given this, we chose to regress power values with memory performance, rather than computing oscillatory power for each level of performance (i.e., zero vs one vs two vs three vs four directions correct) as in the analyses of subsequent memory performance in the main analyses.

For each participant, a trial-based regression on oscillatory power was run, across all sensors (*n* = 274), separately for the first, second, and third encoding trials depending on whether they formed a closed- or open-loop. This analysis was performed on the sensor-, rather than source-level to identify possible time- and frequency-ranges (where the observed fit significantly differed between closed- and open-loops). Beta coefficients were obtained for a given contact x frequency x time point depending on whether they corresponded to the encoding of the first, second, or third pairs of a closed- or open-loop. Here, a positive beta coefficient would indicate that oscillatory power increases with more pairs/directions recalled, while a negative beta coefficient would indicate that power decreases with more pairs/directions recalled. Note that to conserve processing time and capacity, the MEG data was down-sampled from 480 to 100 Hz prior to estimating power.

The beta coefficients were entered into a Monte Carlo cluster permutation test (Maris & Oostenveld, 2007) (*α* = 0.05 (one-tailed), #permutations = 500, minimum ‘neighbourhood’ size = 2), implemented in Fieldtrip (Oostenveld et al., 2011) to identify time- and frequency-ranges where the beta coefficients differed for closed- relative to open-loops. For completeness we performed this analysis for the third as well as the first and second encoding trials (although no differences are expected for the first and second encoding trials). The permutation analyses revealed no significant clusters where the observed fits were consistently greater for closed- vs open-loops for the third encoding trials, nor first or second encoded trials (*ps* > .17). As such, MEG oscillatory activity during the third encoding trial of closed- versus open-loop events did not show the subsequent memory effect for other pairs from that same event that we previously observed in BOLD activity (Horner et al., 2015).
